# Anesthetic Management of a Patient With a Vagal Nerve Stimulator and Poorly Controlled Seizures

**DOI:** 10.7759/cureus.101572

**Published:** 2026-01-14

**Authors:** Lakshmi N Kurnutala, Nickhil Rugnath, Rhea Arora, Ryan Nazari

**Affiliations:** 1 Anesthesiology and Perioperative Medicine, University of Mississippi Medical Center, Jackson, USA; 2 Internal Medicine, Kansas City University, Kansas City, USA

**Keywords:** bis, epilepsy, general anesthesia, status epilepticus, vns

## Abstract

Vagal nerve stimulation (VNS) is an emerging adjunctive therapy for patients with drug-resistant epilepsy. While it can reduce seizure frequency, it rarely results in complete seizure control. It introduces unique challenges in the perioperative setting, including risks of intraoperative seizures, VNS-induced bradyarrhythmias, and respiratory complications.

We present a complex case of a 63-year-old woman with treatment-resistant epilepsy managed with VNS, who underwent open reduction and internal fixation (ORIF) for bilateral femur fractures. On the night before surgery, the patient experienced a tonic-clonic seizure with status epilepticus on the surgical floor, managed with intravenous levetiracetam and midazolam. Despite neurology clearance, intraoperative seizure activity was suspected under general anesthesia, identified by a sudden rise in Bispectral Index (BIS) values. The episode was treated with intravenous levetiracetam, resulting in BIS stabilization and presumed seizure resolution.

This case underscores the heightened perioperative seizure risk in patients with VNS, the importance of continuing antiepileptic therapy, maintaining vigilant intraoperative monitoring, including BIS or continuous electroencephalography (cEEG) monitoring, and selecting anesthetic agents that minimize pro-convulsant potential. The anesthesia team, in collaboration with neurologists, should be well-versed in VNS function, associated complications, and emergency deactivation procedures. This multidisciplinary approach is crucial to optimizing surgical outcomes in patients with refractory epilepsy, providing a comprehensive and reassuring treatment process.

## Introduction

Dr. James Corning (19th century), a neurologist from New York, used an instrument to compress the carotid along with vagus nerve stimulation to treat seizures. Vagal nerve stimulation (VNS) therapy was later introduced in 1988 to evaluate its efficacy in managing epilepsy, and it was not until 1997 that the treatment gained Food and Drug Administration (FDA) approval for the treatment of medically refractory epilepsy [1]. This approval led to the use of VNS in patients who had attempted and failed two or more antiepileptic medications. The VNS system uses a neurocybernetic prosthesis, usually implanted subcutaneously on the left chest, that delivers a pre-set series of electrical impulses to the left vagus nerve via a single lead [1]. Patients can also use a magnet waved over the implanted device to manually deliver stimulation if the patient believes that they may be about to experience a seizure. Newer systems can also detect autonomic changes consistent with seizure prodrome to activate the impulses. Clinical data have suggested that up to 50% of patients with VNS achieve a 50% seizure reduction after 24 months of treatment; however, only a fraction (8%) of those patients report total seizure control [2]. This groundbreaking therapy carries risks, most notably in the surgical and anesthetic settings. Specifically, intraoperative cardiac complications have been encountered, and possible reasons include abnormal electrode placement, unintended collateral stimulation of the cardiac branches, or abnormal vagal anatomy due to individual differences [2,3]. The VNS action on the vagus nerve can additionally lead to respiratory complications through laryngeal dysfunction. As neuromodulation approaches for medication-resistant seizures (VNS, responsive neurostimulation (RNS), and deep brain stimulation (DBS)) become more common, anesthesiologists must be adept in managing these devices and their associated physiological perturbations [3].

The use of VNS in patients undergoing anesthesia poses unique challenges due to the interplay between the seizure activity, anesthetic agents, and device function. Under general anesthesia, particularly with neuromuscular blockade, the classic motor signs of seizures are absent. Without electroencephalographic monitoring, ictal activity can proceed undetected, potentially leading to cerebral metabolic crisis, increased intracranial pressure (ICP), or postoperative neurological deterioration. The anesthetic approach must consider the heightened seizure risk during surgery, often exacerbated by perioperative medication interruptions and dynamic anesthetic drug concentrations [4]. Monitoring strategies such as Bispectral Index (BIS) and continuous electroencephalography (cEEG) are essential for detecting subclinical seizures, which may otherwise go unnoticed during sedation/general anesthesia. VNS involves implanting a device that delivers electrical impulses to the left vagus nerve, reducing seizure frequency in some patients. However, VNS rarely achieves complete seizure control and poses unique challenges in the perioperative setting. We present a case highlighting anesthetic considerations for a patient with VNS and poorly controlled seizures. 

## Case presentation

**Figure 1:**
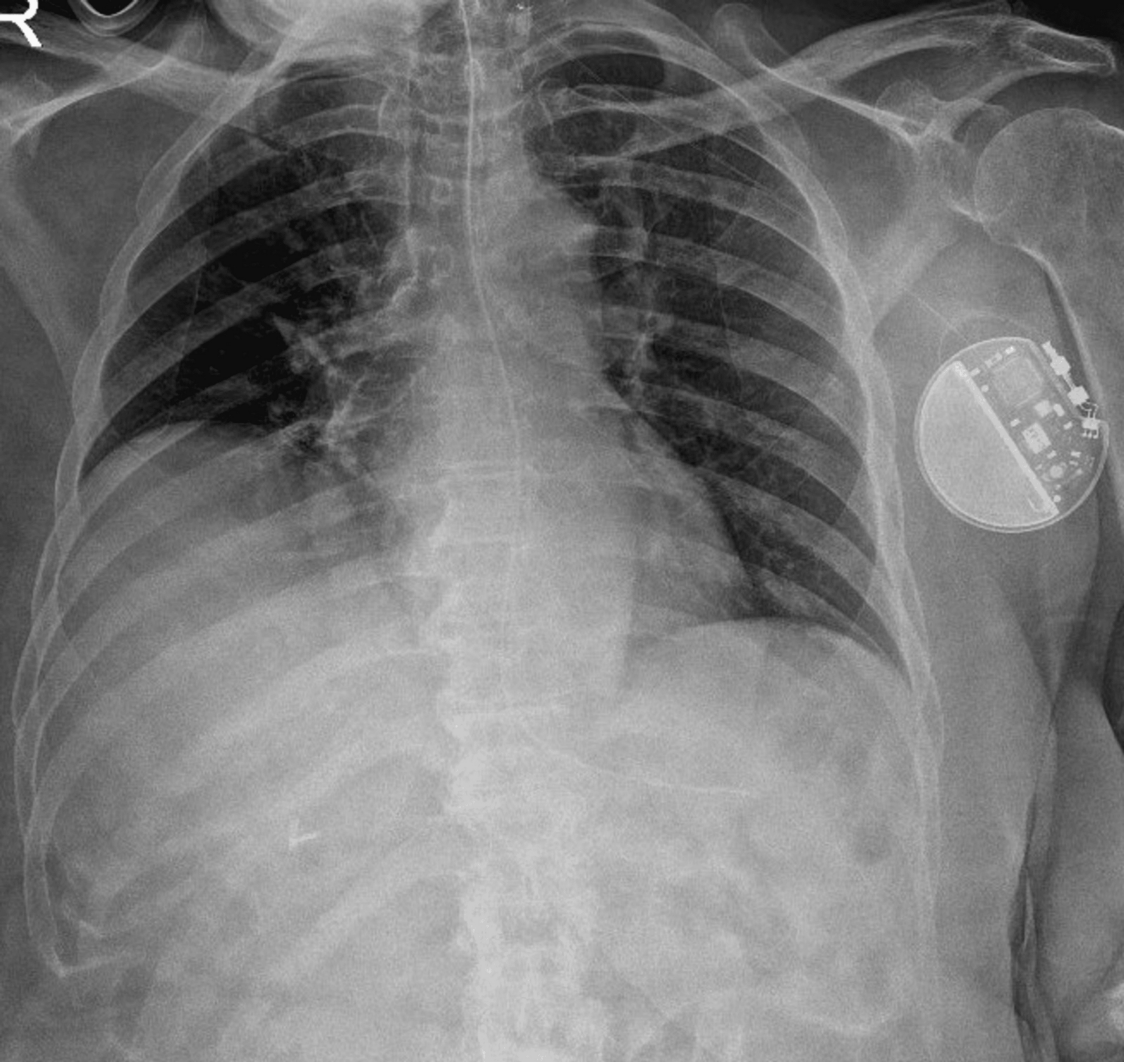
Patient chest X-ray showing the vagal nerve stimulator on the left side of the chest.

A 63-year-old female with a body mass index (BMI) of 37 kg/m² presented with bilateral femur fractures scheduled for open reduction and internal fixation (ORIF). Her past medical history consisted of seizures managed with multiple medications (Depakote oral 1 gm morning/1.5 gm night, brivaracetam 50 mg oral twice daily, felbamate 600 mg oral morning/1200 mg night, and clonazepam 2 mg oral twice daily) and a vagal nerve stimulator (Figure 1). Her additional medical history includes intellectual disability and hypothyroidism. 

On the night before surgery, the patient experienced a tonic-clonic seizure with status epilepticus on the surgical floor as an inpatient. The episode was managed with intravenous levetiracetam (Keppra) and midazolam (Versed), most likely related to not receiving her seizure medications for 24 hours. Her EEG revealed recurrent generalized epileptiform discharge and generalized onset seizures. After achieving seizure control, the patient was evaluated and cleared for surgery with deactivation of VNS for planned surgery by the neurology team.

**Figure 2:**
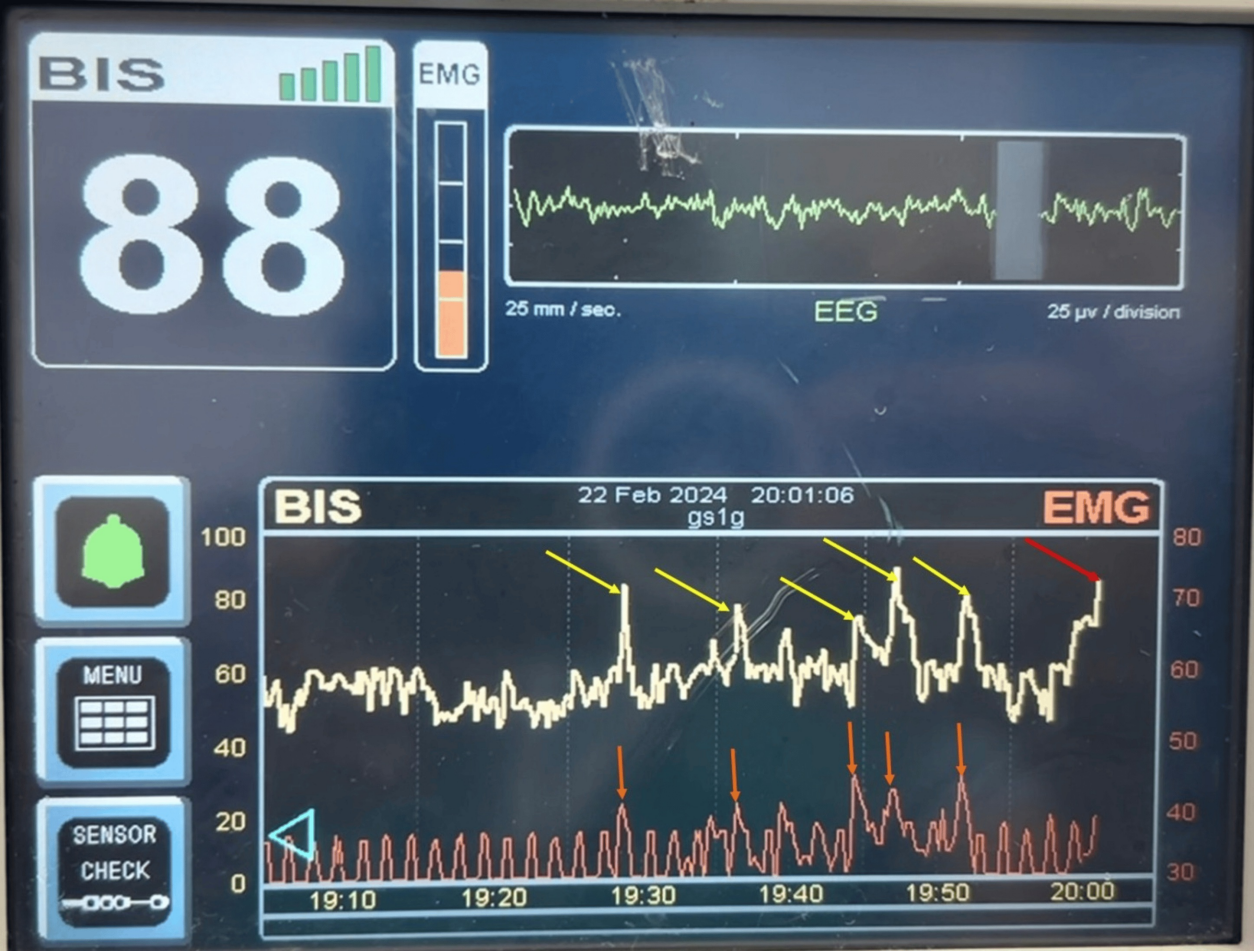
Intraoperative BIS monitoring. Spikes in BIS (yellow arrows) and electromyography (EMG) (orange arrows) indicate the use of electrocautery during surgery. A sudden increase in BIS (red arrow) without a change in EMG indicates increased electrical activity in the brain and the possibility of a seizure. BIS: Bispectral Index

Anesthetic management included general anesthesia (intravenous induction with propofol 120 mg, fentanyl 100 micrograms, lidocaine 50 mg, and rocuronium 50 mg) with endotracheal intubation, American Society of Anesthesiologists (ASA) standard monitors (electrocardiogram (EKG), end-tidal carbon dioxide (EtCO_2_), pulse oximetry (SpO_2_), temperature), neuromuscular monitoring, and BIS monitoring. Intraoperative maintenance includes sevoflurane, oxygen, and an air mixture, with titrated doses of fentanyl for analgesia and rocuronium for muscle relaxation. During the procedure, a sudden rise in BIS scores (from 42 to 88) (Figure 2) without stimulus or electrocautery from baseline suggested seizure activity. There were no major changes in depth of anesthesia, surgical stimulus, or vitals that prompted the administration of 1 gram of intravenous levetiracetam, which subsequently reduced BIS values. 

The patient remained stable for the remainder of the surgical procedure, and the surgery concluded without further events. The patient was extubated at the end of surgery and transferred to the post-anesthesia care unit (PACU) with stable vitals. She did not have any postoperative complications following the surgery, had regular postoperative follow-up with neurology, and continued her home seizure medications.

## Discussion

The presence of VNS in patients undergoing anesthesia requires a comprehensive understanding of seizure control, device function, and complete intraoperative monitoring. Several key factors must be considered to ensure optimal perioperative management.

Seizure detection and monitoring

Patients with poorly controlled epilepsy, despite VNS therapy, remain at risk for perioperative seizures [3,4]. Under general anesthesia, intraoperative seizure activity can go undetected. These cases bring forth the need for cEEG and BIS monitoring. The BIS is a real-time processed EEG parameter that quantifies the depth of anesthesia and the level of consciousness using a numerical scale ranging from 0 to 100. A BIS monitor collects EEG signals and applies algorithms to derive a single value representing cerebral activity. This tool is particularly useful in assessing anesthetic depth, reducing the risk of intraoperative awareness [5]. BIS has also been studied in seizure disorders, as its values can increase or decrease; it was determined that these behaviors could be used to monitor seizures in patients on neuromuscular blockers (NMB), where clinical detection of seizures can be difficult [6]. The BIS scale is as follows: 0: no brain activity, <40: deep hypnosis, 40-60: adequate anesthetic depth for general anesthesia, 60-80: light sedation, with possible recall of intraoperative events, and >80: awake [6].

Although BIS is a valuable tool for monitoring cerebral activity, it is not a direct replacement for continuous EEG, particularly in complex cases of epilepsy. Various factors, including anesthetic agents, cerebral ischemia, hypothermia, and interference with medical devices like electrocautery, can influence BIS values [4,7,8]. Nevertheless, BIS provides a practical alternative and essential addition to traditional cEEG for detecting abnormal cortical activity, guiding intraoperative anesthetic management, and, if necessary, treatment in patients at high risk for seizures [6,8].

Anesthetic agents and seizure control

The choice of anesthetic agents is critical in patients with epilepsy. Certain drugs, such as propofol, thiopental, sevoflurane, and desflurane, have well-characterized anticonvulsant properties [4,7,9]. These agents can be preferred for induction and maintenance. Conversely, agents like ketamine at low doses can lower the seizure threshold and thus facilitate seizures, although at higher doses, ketamine does show anticonvulsant properties [8]. Additionally, maintaining therapeutic levels of anticonvulsant medications is imperative perioperatively. Abrupt cessation of the antiepileptic medication puts the patient at high risk for increased perioperative seizure frequency [4,7-9]. In this patient with a same-day history of status epilepticus, a comprehensive approach to their care is essential, as the literature suggests that the majority of perioperative seizures in patients with a preexisting seizure disorder are likely related to that patient's underlying condition rather than the factors associated with the specific surgery being undergone [9].

VNS-related anesthetic considerations

The VNS device itself can introduce additional perioperative challenges. Electrical stimulation of the vagus nerve has been associated with bradyarrhythmias, necessitating careful cardiac monitoring during anesthesia [1]. Laryngeal dysfunction due to recurrent laryngeal nerve involvement can complicate airway management, particularly during intubation and emergence [1]. Furthermore, external magnets should be readily available to deactivate the VNS in the event of intraoperative arrhythmia or device malfunction [7,10]. New guidelines for anesthetic considerations in VNS patients from 2023 suggest that it may be generally safer to formally deactivate the VNS device altogether just before elective surgery to avoid potential complications of vagal stimulation [10].

## Conclusions

This case emphasizes the importance of vigilant monitoring (BIS, cEEG) for detecting intraoperative seizures in patients with VNS and poorly controlled epilepsy. It is vital to continue antiepileptic medications perioperatively and to select anesthetics with anticonvulsant properties while avoiding seizure-triggering drugs. Anesthesiologists should be familiar with the function of VNS and emergency deactivation protocols. A multidisciplinary approach that integrates anesthesiologists, neurologists, and surgeons is essential for optimizing perioperative outcomes in these high-risk patients.
